# Quality of care for children under two years of age in Brazil’s basic network in 2018: indicators and associated factors

**DOI:** 10.1590/1980-549720230005.2

**Published:** 2023-01-09

**Authors:** Letícia Willrich Brum, Elaine Thumé, Alitéia Santiago Dilélio, Maria del Pilar Flores-Quispe, Nicole Borba Rios Barros, Luiz Augusto Facchini, Elaine Tomasi

**Affiliations:** I Universidade Federal de Pelotas, Programa de Pós-Graduação em Epidemiologia – Pelotas (RS), Brasil.; II Universidade Federal de Pelotas, Faculdade de Enfermagem – Pelotas (RS), Brasil.; III Fundação Oswaldo Cruz, Centro de Integração de Dados e Conhecimentos para Saúde – Salvador (BA), Brasil.; IV Universidade Federal de Pelotas, Faculdade de Medicina – Pelotas (RS), Brasil.; V Universidade Federal de Pelotas, Faculdade de Medicina, Departamento de Medicina Social – Pelotas (RS), Brasil.

**Keywords:** Primary health care, Child care, Health services research, Unified health system, Healthcare disparities, Health inequality monitoring, Atenção primária à saúde, Cuidado da criança, Pesquisa sobre serviços de saúde, Sistema único de saúde, Disparidades em assistência à saúde, Mensuração das desigualdades em saúde

## Abstract

**Objective:**

To evaluate the quality of care for children under two years of age in the primary health care network with data from the external evaluation of the Program for the Improvement of Access and Quality of Primary Care in 2018.

**Methods:**

Users who had children under two years of age who were in the unit at the time of data collection were eligible for the study. The quality of care was evaluated using a synthetic indicator built with questions from the users’ module. The exposure variables were: region, structure of basic health units, and staff process. A univariate analysis was performed and crude and adjusted prevalence ratios were estimated.

**Results:**

The sample was composed of 15.745 users who had children under the age of two years. Only 36.8% (95%CI 36,0–37,6) of users were classified as having received good quality care for their children, with a downward trend in prevalence as the child’s age increased. Better results were observed in the Northeast region, in units that presented all the inputs and vaccines and for teams that used protocols and materials, kept records, performed active search and healthy eating actions.

**Conclusion:**

The prevalence of good quality of care for children under two years of age was low. These data can be useful for managers’ decision-making and for the implementation of actions aimed at professionals, that encourage a higher quality of care to children, mainly the child leaving a consultation with the next appointment scheduled and a first consultation being carried out until their seventh day of life.

## RESUMO

**Objetivo:**

Avaliar a qualidade da atenção a menores de dois anos na rede básica, com dados da avaliação externa do Programa de Melhoria de Acesso e Qualidade da Atenção Básica em 2018.

**Métodos:**

Foram elegíveis para o estudo usuários com filhos menores de dois anos que estavam na unidade no momento da coleta de dados. A qualidade de atenção foi avaliada por meio de um indicador sintético construído com questões do módulo de usuários. As exposições foram: região, estrutura das unidades básicas de saúde e processo de trabalho das equipes. Realizou-se análise univariada e estimaram-se as razões de prevalências brutas e ajustadas.

**Resultados:**

A amostra foi composta de 15.745 usuários que possuíam filhos menores de dois anos. Apenas 36,8% (intervalo de confiança — IC95% 36,0–37,6) dos usuários foram classificados como tendo recebido atenção de boa qualidade para as crianças, com redução das prevalências de acordo com o aumento da idade da criança. Observaram-se melhores resultados para a Região Nordeste, em unidades que apresentaram todos os insumos e vacinas e nas equipes que utilizavam protocolos e materiais, realizavam os registros, a busca ativa e ações de alimentação saudável.

**Conclusão:**

A prevalência de qualidade de atenção a menores de dois anos foi baixa. Os dados podem ser úteis para decisões de gestores e para a execução de ações voltadas para os profissionais, que incentivem maior qualidade de cuidado com a criança, principalmente com relação a, após a consulta, a criança já sair com a próxima marcada e à realização de consulta até os sete dias de vida.

## Introduction

The National Policy for Comprehensive Child Health Care considers monitoring of early childhood by primary health care (PHC) as one of the strategic actions of the axis of promotion and monitoring of growth and development^1^, essential to evaluate the quality of care delivered to children.

Among the determinants of quality of care, the structural characteristics of health services and the work processes of the staff^2^ stand out, based on official protocols^1,3^ that guide PHC, especially in the Family Health Strategy (FHS). This is considered the main model for basic care^4^ and was evaluated through the Program for the Improvement of Access and Quality of Primary Care (PMAQ-AB), which ended in 2019.

Recent approaches have used synthetic indicators for other outcomes^5,6^ that make it possible to assess them separately and combined. Based on PMAQ data, it was identified that the staff’s work process indicators were related to higher prevalences of good quality of care for children under one year old in Brazil compared to structural indicators of basic health units (BHU)^7^. Another study with data from the Northeast reported a higher prevalence of up-to-date vaccination schedule (95.3%) and a lower prevalence of guidance on the best position for the child to sleep (45.7%)^8^.

However, there are still gaps in the measurement of synthetic indicators of the quality of care for children under two years of age in PHC across the country, as well as a need to assess differences according to the child’s age. Knowing these gaps can contribute to the evaluation and planning of health policies and programs in the primary care network, identifying potential weaknesses and strengths. The objective of this study was to evaluate, from the point of view of BHU users, the quality of care delivered to children under two years of age in Brazil, and to invetigate factors related to the structure of establishments and staffs’ work processes.

## METHODS

The PMAQ-AB was implemented in 2011 by Ordinance No. 1654, with the aim to increase access and quality of primary care, one of its components being external evaluation^9^. The program had three cycles: cycle I, from 2011 to 2013; cycle II, from 2013 to 2015; and cycle III, from 2015 to 2019^10^. This is an analytical cross-sectional study with data from the third cycle that took place in 2017 and 2018.

The BHU were selected based on the enrollment of the teams in the PMAQ-AB by the municipal management. Four users were interviewed in each team before the consultations, and those who had used the service in the 12 months prior to the interview or who were not using it for the first time were eligible.

After selecting and training the interviewers, the best routes for displacement were chosen. Previously, municipal managers were contacted to schedule the trip of the teams, who already were acquainted with the instruments to be used. Electronic forms were developed by Universidade Federal do Rio Grande do Norte (UFRN) specifically for this work and applied by researchers by means of tablets. Then, the devices were connected to the internet and the data were sent to the Ministry of Health. The instrument had three modules: observation of the BHU by the interviewers, interview with a health professional about work processes and interview with users^11^.

In this study, only users who had children under two years old were included. We did not use a random sampling process for the selection of teams and users, as the enrollment in the program was done by adhesion. To build the “quality of care” outcome, the following questions were considered:

1. Did the staff conduct an appointment up to seven days after the child’s birth?
2. Is the child up to date on vaccines?
3. Has the child always been consulted by the same health team professionals?
4. After the appointment, is the next one already scheduled?
5. In consultations, was it asked or observed if the child was developing as expected for their age? and
6. Did you receive guidance on feeding the child up to two years old?

For the outcome, a synthetic indicator of quality was constructed based on the sum of positive responses, with each respondent being able to choose a score from 0 to 6. Afterwards, this indicator was dichotomized and good quality care was considered as referred by users who gave affirmative answers to the six questions.

As exposure variables, the region (North, Northeast, Midwest, South and Southeast) was considered for the municipalities; for the BHU, the availability of at least one item of a set of inputs and vaccines was observed; as for the staff, the use of different protocols and materials, ways of recording, active search and food promotion actions were observed. For the structure of the health units and the staffs’ work processes, synthetic indicators were created with the total number of affirmative answers to each of the six items surveyed, two relating to structure and four relating to work processes (Table 1).

**Table 1:** Sample distribution according to structural characteristics of basic health units and work process of the teams providing care to children under two years of age. Brazil, Program for Improvement of Access and Quality of Primary Health Care: 2018.

Characteristics	Yes	
	n	%
Available supplies		
Needles and Syringes	15,545	98.7
Children’s blood pressure device in usable condition	12,344	78.6
Children’s stethoscope (in usable condition)	10,909	69.5
Nebulizer device	12,802	81.5
Children’s scales in usable condition	15,220	96.9
Children’s anthropometric rulers in usable condition	14,517	92.4
Stretchers/tables for clinical examination in usable condition	15,630	99.5
Vaccine-exclusive refrigerators in usable condition	12,032	76.6
Autoclaves in usable condition	12,435	79.2
Clinical thermometers in usable condition	15,402	98.1
Child health booklet	12,587	80.1
Vaccination card/proof (always available)	14,623	93.1
All items	4,798	30.5
Vaccines Always available:		
Hepatitis A	13,540	95.2
Hepatitis B	13,740	96.6
Meningococcal C	13,344	93.9
Poliomyelitis 1, 2 and 3 (attenuated) (VOP)	12,838	90.3
Poliomyelitis 1, 2 and 3 (inactivated) (VIP)	13,591	95.6
Pneumococcal 10	13,583	95.5
Tetravalent or triple viral	13,589	86.3
Pentavalent or triple bacterial	13,683	86.9
Oral human rotavirus vaccine	12,393	87.2
All items	10,667	75.0
Protocols and materials used by the staff:		
Focused on children under two years of age	14,269	92.5
Updated registration of children up to two years of age in the territory	14,489	93.9
Child health booklet for follow-up	15,354	97.7
Copy of equivalent child health booklets	13,544	86.2
All items	12,222	79.2
Staff keeps track of:		
Vaccination	15,384	97.9
Growth and development	15,224	96.9
Nutritional status	15,065	95.9
Foot test	14,750	93.9
Domestic violence	12,213	77.7
Accidents	11,936	76.0
All items	11,109	70.7
Staff conducts an active search for:		
Premature children	14,655	93.3
Underweight children	14,981	95.4
Children with delayed childcare consultation	14,512	92.4
Children with late immunization schedule	15,194	96.7
All items	13,806	87.9
Staff develops actions of:		
Promotion of exclusive breastfeeding for children up to six months	15,444	98.3
Encouraging of introduction of healthy foods and continued breastfeeding from six months of age onwards	15,433	98.2
Compliance with The Brazilian Code for Marketing of Infant and Toddler’s Food, Teats, Pacifiers and Baby Bottles (NBCAL)	13,780	87.7
All items	13,617	86.7

The Stata 16.0^12^ package was usedfor data analysis. First, a univariate analysis was performed, considering the χ² test for heterogeneity of nominal dichotomous and categorical variables and the χ² test for trends for ordinal categorical variables. The outcome was also stratified according to children’s age group (in months). Poisson regression was used, with robust variance^13^, in a hierarchical analysis model^14^, to estimate crude and adjusted prevalence ratios. The first level included the region, the second included synthetic indicators of the units’ structure, the third included the synthetic indicators of work processes, and the fourth included the age group of the children in months (0–6, 7–12, 13–18, 19–24). The value of p<0.05 was determined as statistically significant in the association analyses.

The study was approved by the Research Ethics Committee of Universidade Federal de Pelotas, under Protocol 2,453,320. All participants signed the Free and Informed Consent Form.

## RESULTS

From across the national territory, 28,939 BHUs and 37,350 staffs were included in the sample. About four users were interviewed on each team, totaling 140,444. The sample consisted of 15,745 users who had children under two years of age, corresponding to 11.2% of the total number of respondents during PMAQ Cycle III. The number of losses and refusals was not made available by the Ministry of Health. Higher proportions of users were found in municipalities in the Northeast (36.4%) and Southeast (34.2%). The South, North and Midwest regions had prevalence values of 11.1%, 9.4% and 9.0%, respectively.

Considering the structure of BHU, the listed inputs were present in more than 70% of the services, but only 30.5% of them had all of them. Three-quarters of the services had all the necessary vaccines available (Table 1).

Almost all teams carried out childcare consultations, more than 85% of the them used certain protocols and materials necessary for the care of children, but only 79.2% had them all available. With regard to follow-up records, the frequencies were greater than 75%, but 70.7% of the teams made all the records. More than 90% of the teams reported carrying out active searches separately for groups of children, and 87.9% stated carrying out all searches. Two of the three items investigated on healthy eating promotion were cited by 98% of the teams and 86.7% of them mentioned all items (Table 1).

Most of the synthetic indicators showed prevalence values greater than 80%, except consultations within seven days of life (64.0%) and the child leaving the consultation with the next one scheduled (63.3%) (Table 2). The prevalence of quality of care—taken as an outcome here—was only 36.8% in the sample (confidence interval—95%CI 36.0–37.6), with a significant downward trend as the child’s age increased (Table 2). Higher prevalence values of this outcome were found in the Northeast Region (40.2%), in BHUs that had all supplies (65.3%) and vaccines (38.6%) available, teams that followed protocols and had and used all the necessary materials (39.5%), who kept records appropriately (39.0%), performed active searches (38.1%), and promoted healthy eating actions (38.1%) (Table 3).

**Table 2:** Age distribution, variables of quality of care indicator and quality of care according to age of children under two years of age in primary health care. Program for Improvement of Access and Quality of Primary Health Care: 2018 (n=15,745)

Variable (n)	%
Age (months) (15,695)	
0–6	37.0
7–12	22.3
13–18	19.4
19–24	21.3
The child consults up to 7 days of life (15,430)	64.0
The child’s vaccines are up to date (15,701)	96.1
Always consulted with the same team of professionals (14,726)	80.2
They leave consultations with the next appointment scheduled (14,645)	63.3
In consultations, it was asked or observed whether the child was developing as expected for their age (14,692)	90.7
Received guidance on feeding the child up to two years old (15,393)	85.0
All variables (quality of care) (13,997)	p<0.001*
0–6 months	40.2
7–12 months	37.7
13–18 months	35.0
19–24 months	32.0
All age groups	36.8

*Trend χ^2^.

**Table 3:** Crude and adjusted prevalence ratios for quality of care for children under two years of age according to exposures. Program for Improvement of Access and Quality of Primary Health Care: 2018 (n=15,745).

Variable	% outcome	p-value	Crude PR (95%CI)	Adjusted PR (*) (95%CI)
Region				
North	24.2	<0.001	1.0	1.0
Northeast	40.2		1.66 (1.50–1.84)	1.66 (1.50–1.84)
Midwest	30.2		1.25 (1.10–1.42)	1.25 (1.10–1.42)
Southeast	38.3		1.58 (1.43–1.76)	1.58 (1.43–1.76)
South	36.9		1.52 (1.36–1.71)	1.52 (1.36–1.71)
Supplies				
No	34.8	<0.001	1	1
Yes	41.4		1.19 (1.14–1.34)	1.13 (1.08–1.18)
Vaccines				
No	34.1	<0.001	1	1
Yes	38.6		1.13 (1.07–1.20)	1.12 (1.06–1.18)
Protocols and materials				
No	27.2	<0.001	1	1
Yes	39.5		1.45 (1.36–1.55)	1.24 (1.15–1.34)
Records				
No	31.5	<0.001	1	1
Yes	39.0		1.24 (1.18–1.30)	1.09 (1.03–1.15)
Active searches				
No	26.8	<0.001	1	1
Yes	38.1		1.42 (1.31–1.55)	1.15 (1.05–1.27)
Healthy eating promotion actions				
No	27.9	<0.001	1	1
Yes	38.1		1.37 (1.26–1.48)	1.15 (1.06–1.26)
Age (months)				
0–6	40.2	<0.001*	1	1
7–12	37.7		0.94 (0.89–0.99)	0.95 (0.89–1.00)
13–18	35.0		0.87 (0.82–0.92)	0.88 (0.83–0.94)
19–24	32.0		0.80 (0.75–0.85)	0.83 (0.78–0.88)

PR: prevalence ratio; CI: confidence interval. *Level 1: region; Level 2: level 1 + Basic Health Units structure indicators; Level 3: levels 1 and 2 + indicators of the staff’s work process; and Level 4: Levels 1, 2 and 3 + the child’s age group.

In both crude and adjusted analyses, all variables had a statistically significant association with the outcome (Table 3). The prevalence ratio (PR) found in the Northeast Region (PR 1.66; 95%CI 1.50–1.84) was higher compared to the North Region. Quality of care showed higher prevalence values in the BHUs that had all supplies (PR 1.13; 95%CI 1.08–1.18) and all vaccines available (PR 1.12; 95%CI 1.06–1.18), in teams that used all protocols and materials (PR 1.24; 95%CI 1.15–1.34), kept appropriate records (PR 1.09; 95%CI 1.03–1.15), performed active searches (RP 1 .15; 95%CI 1.05–1.27) and all actions to promote healthy eating (PR 1.15; 95%CI 1.06–1.26). As age increased, there was a significant decrease in the quality of care.

## DISCUSSION

Our study identified a low prevalence of good quality of care for children under two years of age in primary care across the country, with marked differences according to region, structure of BHU and work processes of the teams. In addition, the prevalence of quality decreased as the child’s age increased.

Higher quality was found in the Northeast and Southeast regions. No studies that evaluated the quality of care for children under two years of age in Brazil as a whole were found, only research comparing the states of the Northeast Region^8^, in which the authors evaluated indicators separately. Other studies that evaluated the quality of PHC in different groups with PMAQ data—such as pregnant women and people with chronic diseases—also found an association between quality and region, with better care indicators in the Southeast Region^5,15^. The better performance in the Southeast might stem from better structure of services and care conditions in the municipalities that also accumulate better socioeconomic indicators^16^. With regard to the Northeast, it is estimated that the greater FHS coverage, combined with its successful history in the region, manages to keep the indicators at high levels despite the socioeconomic vulnerability of most municipalities^16,17^.

The quality of care was higher in health units that had all the necessary vaccines and supplies for child care, basic structural components for PHC^4^, as it is believed that the units that have all the supplies can provide better care to users. When evaluating the BHU census in cycle I of the PMAQ-AB, only 4.8% of the units reached the maximum evaluation score based on type of team, list of professionals, operating shifts, available services, facilities and inputs^18^. When verifying the presence of equipment, materials and inputs in cycle III of the PMAQ-AB, most prevalence values were greater than 90.0%. As for vaccines, most immunobiological assets had a prevalence of less than 95.0%, except for the hepatitis B vaccine (95.7%)^10^. Also with data from the PMAQ, a study identified that, despite an increase in prevalence between 2012 and 2014, low levels were recorded regarding adequate structure of materials and medicines for the care of people with diabetes^6^.

According to Donabedian, better results are obtained by adequate work process, present in more robust structures^2^. Our study shows that, as more organized teams, which followed protocols, had and used the necessary materials, kept all records, conducted an active search and promoted healthy eating actions had better performance in the synthetic quality indicator. However, it should be noted that none of these indicators was greater than 90.0%, which reflects a need to encourage the best work process by the teams, since the practices evaluated depend almost exclusively on the action of professionals. In Brazil, PHC has protocols to support the actions of health professionals, with emphasis to PHC notebooks numbers 23^19^ and 33^3^, which list necessary routines and conducts. Some aspects related to job dissatisfaction cited by FHS professionals are the lack of materials, inadequate physical structure, and lack of qualification of the teams^20^, estimated to be the reason for the lack of actions for the care of children under two years old. The essential attributes listed by Starfield^21^ with the highest prevalence present in BHUs across Brazil were first contact with users and comprehensiveness, however longitudinal actions had the lowest prevalence^22^.

Higher quality was found for children aged zero to six months, with a downward trend as age increased, pointing to the need to extinguish these differences. Although there are recommendations on nutrition for children aged up to six months^23^ and also other procedures^3^, there is also a need to care for other age groups—which made up 63% of the sample—, including complementary feeding, continued evaluation of growth and development, and proper vaccination according to schedule.

One of the limitations of the study is that professionals’ responses may have been overestimated, considering that they were previously familiar with the instrument and could have better prepared services for external evaluation, especially in terms of structure. The interviews with users at the units may also have been influenced by the staff members, which was minimized by the fact that they answered the questionnaire before the consultations. Another limitation may be related to the scope of the questions available in the instrument, namely the lack of information on the assessment of food consumption and on the questioning of professionals to users about difficulties and queries regarding child care.

The strengths of our study were the national coverage of the sample, which reached almost 100% of the existing teams in the period, the construction of a synthetic indicator for quality of care for children under two years of age, and the investigation of characteristics of municipalities, services and staffs in a hierarchical model with adjusted measures.

These data available to managers will be useful to support decisions regarding the improvement in the structure of health units and in the qualification of professionals, via continued education programs. Our findings also serve as a basis for carrying out actions that seek greater encouragement to the quality of care for children by professionals. In addition, the results of this study are expected to contribute to the continuity of investigations on the quality of care for children under two years of age in primary care.
